# Relationship between salt consumption and iodine intake in a pediatric population

**DOI:** 10.1007/s00394-020-02407-w

**Published:** 2020-10-21

**Authors:** Roberto Iacone, Paola Iaccarino Idelson, Angelo Campanozzi, Irene Rutigliano, Ornella Russo, Pietro Formisano, Daniela Galeone, Paolo Emidio Macchia, Pasquale Strazzullo, The MINISAL-GIRCSI Study Group

**Affiliations:** 1 Department of Clinical Medicine and Surgery, Federico II University of Naples Medical School, Naples, Italy; 2 Pediatrics, Department of Medical and Surgical Sciences, University of Foggia Medical School, Foggia, Italy; 3 Pediatrics, IRCCS Casa Sollievo Della Sofferenza, San Giovanni Rotondo, Foggia, Italy; 4 Translational Medical Science, Federico II University of Naples Medical School, Naples, Italy; 5 Italian Ministry of Health, Center for Disease Prevention and Control, Rome, Italy

**Keywords:** Iodine prophylaxis, Iodine deficiency disorders, Thyroid, Salt restriction, Hypertension, Pediatric age, Iodine intake, 24 h urinary excretion

## Abstract

**Purpose:**

The World Health Organization recommends reduction of salt intake to < 5 g/day and the use of iodized salt to prevent iodine deficiency states. A high prevalence of excess salt consumption and an inadequate iodine intake has been previously shown in an Italian pediatric population. It was appropriate, therefore, to analyse in the same population the relationship occurring between salt consumption and iodine intake.

**Methods:**

The study population was made of 1270 children and adolescents. Estimates of salt consumption and iodine intake were obtained by measuring 24 h urinary sodium and iodine excretion.

**Results:**

The iodine intake increased gradually across quartiles of salt consumption independently of sex, age and body weight (*p* < 0.001). Median iodine intake met the European Food Safety Authority adequacy level only in teenagers in the highest quartile of salt consumption (salt intake > 10.2 g/day). We estimated that approximately 65–73% of the total iodine intake was derived from food and 27–35% from iodized salt and that iodized salt made actually only 20% of the total salt intake.

**Conclusion:**

In this pediatric population, in face of an elevated average salt consumption, the use of iodized salt was still insufficient to ensure an adequate iodine intake, in particular among teenagers. In the perspective of a progressive reduction of total salt intake, the health institutions should continue to support iodoprophylaxis, in the context of the national strategies for salt reduction. In order for these policies to be successful, in addition to educational campaigns, it is needed that the prescriptions contained in the current legislation on iodoprophylaxis are made compelling through specific enforcement measures for all the involved stakeholders.

**Electronic supplementary material:**

The online version of this article (10.1007/s00394-020-02407-w) contains supplementary material, which is available to authorized users.

## Introduction

Iodine is essential for the synthesis of thyroid hormones, which are pivotal for a healthy growth [1] and a normal neurological development. In children, long-term inadequate iodine intake causes insufficient cognitive development, including a low intelligence quotient [2, 3]. In many regions of the world, the amount of iodine taken up with food is not sufficient to fulfil the physiological needs and to achieve an adequate intake. Thus, the preferred strategy suggested by the World Health Organization (WHO) for preventing iodine insufficiency is the fortification of food-grade salt with iodine [4, 5]. Salt is considered an appropriate vehicle for fortification with iodine, because it is widely consumed, salt iodizing technology is well standardized and inexpensive, and the addition of iodine to salt does not affect its taste or smell. WHO also recommends reduction of salt intake to less than 5 g/day [6] in order to prevent arterial hypertension and the increased risk of stroke, coronary heart disease, and premature death [7, 8]. According to WHO, a concentration of iodine in the iodized salt of between 20 and 40 mcg/g should allow an adequate iodine intake even at a salt intake of 5 g/day or below [9]. The Italian law 55/2005 introduced measures aimed to promote the consumption of iodine-enriched salt throughout the national territory. The amount of iodine added to food-grade salt (30 mcg iodine/g of salt) was supposed to allow an adequate iodine intake [10] for the Italian population Since 2009, the National Observatory for the Monitoring of Iodoprophylaxis in Italy (OSNAMI) has been established at the Istituto Superiore di Sanità for the surveillance of the National program of iodoprophylaxis. Between 2011 and 2012, the percentages of iodized salt sold for household use and for the catering system were, respectively, 50% and 23% according to the Italian Ministry of Health [10]. However, a survey carried out by our group in a national sample of Italian school children and adolescents in 2012 [11, 12] showed that a sizable proportion of this young population, especially girls, had a lower than adequate iodine intake despite having a salt intake greater than that recommended by WHO. Thus, the present study aimed to analyse the relationship between salt consumption and iodine intake in the same population and to estimate the actual contribution made by the use of iodized salt to iodine intake.

## Subjects and methods

### Study population

Dietary iodine intake and salt consumption were assessed in a nationwide sample of 1270 Italian children and adolescents recruited with the collaboration of the Italian Society for Pediatric Gastroenterology, Hepatology and Nutrition (SIGENP) in the framework of the MINISAL-GIRCSI Program in 2012 [13, 14]. Participants aged 6–17 years were enrolled in 10 Italian regions representative of the whole Italian territory (Northern Italy: Piemonte, Lombardia, Emilia Romagna; Central Italy; Toscana, Umbria, Marche, Lazio; Southern Italy: Puglia, Campania, Calabria) and provided a 24-h urine collection. Urine collection, as well as demographic and anthropometric data, took place from January to June 2012. Unfortunately, no information could be collected about the habitual diet and other lifestyle habits. The estimate of iodine intake and salt consumption was made by measuring iodine and sodium excretion using 24 h urine collections. A detailed description of the study participants' recruitment methods and the procedures used for the collection of demographic and anthropometric data, as well as of the biological samples, has been previously reported [11, 12]. Briefly, one thousand two hundred and seventy healthy participants aged 6–17 years, whose parents or legal tutor agreed to let them participate in the study, performed a 24-h urinary collection and underwent a standard physical examination, and an anthropometric evaluation, including measurement of height and weight and calculation of body mass index (BMI) and BMI Z-score, as previously described [11, 12].

### Protocol for the estimation of sodium and iodine intake

Each participant (or his/her caregiver, in case of younger children) received a plastic container for the 24-h urine collection, together with detailed instructions on how to collect complete 24-h urines. Recommendations were made to void in the morning after rising and to discard urine from the first micturition completely, noting the time of voiding as the start time of the urine collection, and to collect all the urines produced during the following 24 h, including the first void of the following morning. Once the collection was returned, the total volume of the urine was recorded, and two samples were extracted after shaking. The samples were immediately stored in plastic containers and frozen at − 30 °C for later analyses. Iodine and creatinine measurements were carried out, respectively, at the Departments of Clinical Medicine and Surgery and of Translational Medical Science, at Federico II University of Naples Medical School. Urine iodine concentration (UIC) was analyzed as previously described [12] by an Autoanalyzer 3 system (Bran + Luebbe GmbH), using the ceric-arsenious acid reaction and digestion method by ultraviolet irradiation. Urinary sodium concentration was measured by ion-selective electrode potentiometry [11] using an ABX Pentra 400 apparatus (HORIBA ABX, Rome, Italy). Urinary iodine excretion was expressed as micrograms per 24 h. Dietary iodine intake was estimated with the formula:

$${\text{Daily iodine intake }}\left( {{\text{mcg}}/{\text{day}}} \right)\, = \,{{\text{Urinary iodine excretion}} \mathord{\left/ {\vphantom {{\text{Urinary iodine excretion}} {0.92}}} \right. \kern-\nulldelimiterspace} {0.92}}$$

Assuming that 92% of the consumed iodine is excreted in the urine [15, 16]. The prevalence of inadequate iodine intake in the study population and in subgroups thereof was assessed according to the EFSA adequate intake (AI) values for iodine [16] (i.e., 90 mcg/day for children aged 4–10 years, 120 mcg/day for children aged 11–14 years, and 130 mcg/day for adolescents). Urinary sodium excretion was expressed as moles per 24 h. Daily salt consumption was estimated assuming that 90% of the sodium ingested is excreted in the urines [17]. Thus

$${\text{Daily salt consumption }}\left( {{\text{grams}}/{\text{day}}} \right)={{{\text{Urinary sodium excretion }} \times { 58}{\text{.44}}} \mathord{\left/ {\vphantom {{{\text{Urinary sodium excretion }} \times { 58}{\text{.44}}} {0.90}}} \right. \kern-\nulldelimiterspace} {0.90}},$$

where 58.44 is the NaCl molar mass.

The prevalence of excess sodium (salt) consumption in the study population was assessed based on the maximum daily salt intake recommended by the World Health Organization (i.e., < 2 g sodium/day, equivalent to 5 g salt/day) [6].

Urinary creatinine, measured by the kinetic Jaffé reaction using an ABX Pentra 400 apparatus (HORIBA ABX), was used as an indicator of the adequacy of the 24-h collection. Participants were excluded from the analysis if they reported not having provided complete urine collections or presented a urinary creatinine excretion < 0.1 mmol/kg body weight, corresponding to the fifth percentile of the 24-h urinary creatinine distribution in a population of healthy individuals aged 3–18 years [18]. Quality controls were performed using the low and high Urichem Gold Standards from Bio Development SRL, Milan, Italy.

### Estimation of iodine intake derived from food and of iodized salt consumption

The fraction of daily iodine intake presumably coming from foods for a population having age and gender distribution similar to our study population has been estimated using data of the Italian National Food Consumption survey (INRAN-SCAI) [19] and the WHO table for the mean iodine content of different foods [20]. The mean iodine content of different foods (mcg/100 g) was multiplied for the respective levels of consumption (g/day). Food consumption data are provided separately for male and female teenagers, and combined for children 3–9 years, according to the INRAN-SCAI survey (19). The estimate of total iodine provision by food and by iodized salt was made considering a 30% iodine loss occurring through cooking and stocking [21]. The iodine intake from food was also expressed as iodine/1000 kcal using the INRAN-SCAI database of nutrients and energy intake in the Italian population [22].

The amount of iodine deriving from the use of salt was estimated by subtracting the iodine provided by food from the total iodine intake (estimated from 24 h urinary iodine excretion). The amount of iodized salt (g/day) consumed was estimated by dividing the iodine intake from salt by 21 (the micrograms of iodine within 1 g of iodized salt after 30% loss though cooking and stocking). The use of iodized salt was expressed as a percentage of the total salt intake.

### Statistical analysis

Statistical analysis was performed using the Statistical Package for the Social Sciences (SPSS Statistics for Windows, Version 22.0; IBM Corp, Armonk, NY, USA). The descriptive statistics covered the whole study population and the population stratified by gender and by quartiles of salt intake. As all the numeric variables under investigation did not follow a Gaussian distribution (as assessed by Kolmogorov–Smirnov test), the results were expressed as median and interquartile range. For categorical variables, the results were reported as frequencies (%). Non-parametric tests were used to test between-group differences (Mann–Whitney test in the case of two groups and Kruskal–Wallis test for more than two groups). The Jonckheere–Terpstra test was used for the analysis of the trend and Spearman rank correlation analysis to evaluate the possible associations among the variables under investigation. Two-sided p values less than 0.05 were considered statistically significant.

## Results

**Fig. 1:**
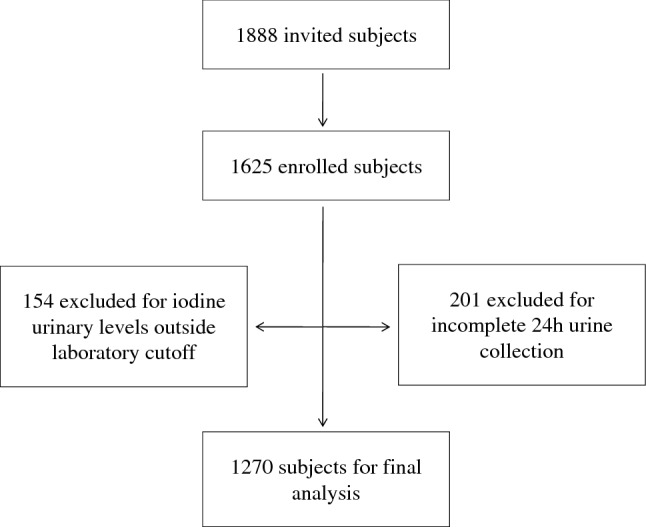
Flowchart of the study population

From an initial number of 1888 invited subjects, 1625 were enrolled (86%). 201 subjects were excluded from the analysis because of suspected incomplete 24-h urine collection, based on the criteria indicated in Methods. A further 130 subjects were excluded, because their UIC was below the analytical sensitivity of the method (5 μg/L), and 24 subjects were excluded, because their UIC was > 400 μg/L, presumably ascribable to the occasional consumption of food with very high iodine content (e.g., fried kelp) [23] or more likely to non-dietary iodine absorption from other sources such as topical antiseptic, disinfectants or iodine-containing toothpaste. Thus, the analysis was eventually performed on 1270 subjects (Fig. 1).

Table 1 shows median and interquartile range (IQR) for age, BMI, salt consumption, and iodine intake of boys (*n* = 677), girls (*n* = 593) and whole study population. The overall medians and IQRs of iodine concentration and urinary volumes were 111 (62–186) mcg/L and 900 (700–1203) ml/24 h, respectively. Overall, there were 78.5% (997/1270) of study participants with a salt consumption greater than 5 g/day and 50.6% (643/1270) of participants with an iodine intake below the age specific EFSA AI for iodine [16]. There were no significant differences with regard to age and BMI between boys and girls. The fraction of participants that exceeded 5 g of salt per day was 75.9% (450/593) for girls and 80.1% (547/677) for boys; chi-square = 4.52, *p* = 0.033. Female participants with iodine intakes lower than the EFSA AI for age were 343/593 (57.8%), whereas male participants were 300/677 (44.3%); chi-square = 23.15, *p* < 0.001).

**Table 1:** Medians (IQR) of age, BMI, salt consumption and iodine intake in the overall study population and by gender

	Overall *n* = 1270	Boys *n* = 677	Girls *n* = 593	p*
Age, y	10.0 (7.8–12.3)	10.0 (7.8–12.0)	10.1 (8.0–12.3)	0.314
Body weight, Kg	38.0 (29.0–50.0)	38.0 (29.0–50.0)	38.0 (29.3–49.8)	0.885
BMI *Z* score	0.67 (− 0.12–1.44)	0.68 (− 0.10–1.50)	0.66 (− 0.21–1.39)	0.149
Salt intake, g/24 h	7.5 (5.4–10.2)	7.9 (5.6–10.8)	7.0 (5.0–9.5)	< 0.001
Iodine intake, mcg/24 h	109 (63–168)	118 (71–178)	97 (54–151)	< 0.001

^*^Mann–Whitney *U* test for independent samples (male vs. female participants)

Salt intake and iodine intake were directly associated (Spearman rho = 0.333; *p* < 0.001) and salt intake variation explained approximately 11% of the variation in iodine intake. Salt intake was also related to age (Spearman rho = 0.312, *p* < 0.001), body weight (Spearman rho = 0.362, *p* < 0.001), and weakly to BMI *Z* score (Spearman rho = 0.085, *p* = 0.003). Iodine intake was weakly associated with age (Spearman rho = 0.119, *p* < 0.001), body weight (Spearman rho = 0.125, *p* < 0.001), but not with BMI *Z* score (Spearman rho = 0.015, *p* = 0.604). Nevertheless, partial correlation analysis indicated that the direct association between salt consumption and iodine intake was largely independent from sex, age and body weight (Spearman rho = 0.297, *p* < 0.001).

**Fig. 2:**
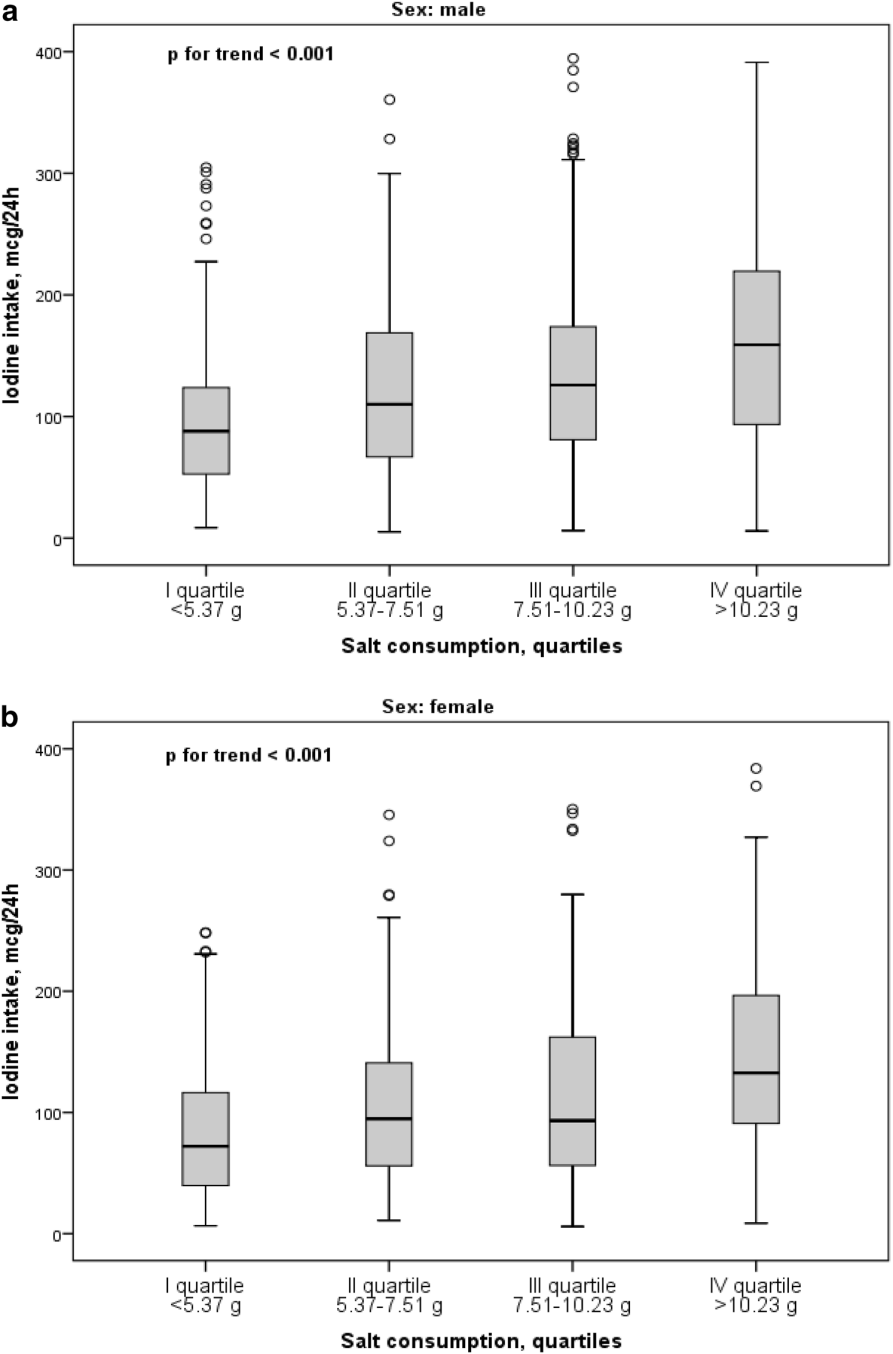
Shows box-and-whisker plots with outliers of iodine intake (mcg/24 h) by quartiles of salt consumption. The box is drawn from I to III quartile with a horizontal line drawn in the middle to denote the median. The top whiskers show a distance of 1.5 times the interquartile range (IQR) measured out of the higher quartiles. The outliers are the observed points from the dataset that fall outside of that distance. The bottom whiskers indicate the minimum values, as no case has a value at a distance of 1.5 times of the IQR measured out of the lower quartiles. **a** Iodine intake median (IQR) values for boys were: I quartile 86 (50–123), *n* = 155; II quartile 110 (62–169), *n* = 151; III quartile 128 (81–177), *n* = 178; IV quartile 161 (95–230), *n* = 193; *p* for trend < 0.001 (Jonckheere–Terpstratest), *p* of Kruskal–Wallis test for independent samples < 0.001. **b** Iodine intake median (IQR) values for girls were: I quartile 71 (39–116), *n* = 163; II quartile 95 (60–142), *n* = 166; III quartile 93 (56–165), *n* = 140; IV quartile 134 (94–202), *n* = 124; *p* for trend < 0.001 (Jonckheere–Terpstratest), *p* of Kruskal–Wallis test for independent samples < 0.001

Upon stratification of the study population by quartile of salt consumption, a progressive increase in iodine intake was apparent from the first to the fourth quartile (Fig. 2) with a significant trend in both male and female participants (p for trend < 0.001). Medians (IQRs) of iodine intake for male participants (Fig. 2a) were: first quartile of salt consumption 86 (50–124), second quartile 110 (65–169), third quartile 127 (81–178), fourth quartile 161 (95–230). Medians (IQR) of iodine intake for female participants (Fig. 2b) were: first quartile of salt consumption 71 (39–116), second quartile 95 (56–142), third quartile 93 (56–165), fourth quartile 133 (94–203).

Median values of iodine intake according to degree of salt consumption and by age category are provided in Table 2. Iodine intake gradually increased with increasing salt consumption in all age groups. With reference to the EFSA AI values for iodine (90 mcg/day for the age group 4–10 years, 120 mcg/day for the age group 11–14 years and 130 mcg/day for age 15–17 years), median iodine intake was below the adequacy level in all age groups for participants in the first quartile of salt consumption and in the age groups 11–14 and 15–17 y for participants in the second and third quartile. Iodine intake met the adequacy level in all age groups only for participants in the highest quartile of salt consumption.

**Table 2:** Iodine intake (mcg/d) and salt consumption (g/d) by quartiles of salt intake and age groups, median (IQR)

	I quartile (< 5.37 g)	II quartile (5.37–7.51 g)	III quartile (7.51–10.23 g)	IV quartile (> 10.23 g)	*p* for trend*
Age 6–10 y	*n* = 235	*n* = 210	*n* = 190	*n* = 127	
Salt consumption	4.1 (3.1–4.8)	6.4 (5.9–7.0)	8.4 (7.9–9.3)	12.0 (11.1–13.9)	< 0.001
Iodine intake	75^#^ (46–119)	97 (58–146)	112 (69–170)	142 (87–218)	< 0.001
Age 11–14 y	*n* = 66	*n* = 92	*n* = 98	*n* = 145	
Salt consumption	3.8 (3.1–4.7)	6.4 (6.0–7.0)	8.7 (8.1–9.4)	12.6 (11.3–15.0)	< 0.001
Iodine intake	87^#^ (46–133)	117^#^ (62–177)	117^#^ (73–173)	159 (106–239)	< 0.001
Age 15–17 y	*n* = 17	*n* = 15	*n* = 30	*n* = 45	
Salt consumption	4.2 (3.5–4.6)	6.8 (5.7–7.1)	8.8 (8.2–9.5)	12.4 (11.1–15.6)	< 0.001
Iodine intake	46^#^ (40–84)	117^#^ (49–139)	115^#^ (62–203)	135 (81–199)	< 0.001

^*^Jonckheere–Terpstra test for trend

^#^The hashtag denotes a median value below the EFSA Adequate Iodine Intake (6–10 years, 90 mcg/day; 11–14 y, 120 mcg/day; 15–17 y, 130 mcg/day)

Iodine in our study population could derive either from food or from iodized salt. Since we lacked direct information about iodine intake from food, we estimated this amount by multiplying the average iodine content of foods [20] by the respective age and sex specific food consumption of the Italian children and adolescent population, as described in Methods and in the supplementary tables. The estimated iodine provision by foods referred to energy intake was 44 mcg iodine/1000 k cal for children, 45 mcg iodine/1000 kcal for female teenagers and 35 mcg iodine/1000 kcal for male teenagers. Table 3 reports the estimates for the average iodine intake from foods and from iodized salt and the amount and proportion of iodized salt consumption in the study population for children below 10 y and for male and female teenagers.

**Table 3:** Estimates of iodine intake from foods and salt, and of iodized salt intake as absolute and percentage values by age group, mean ± SD for salt and iodine intake, and arithmetic mean for the other variables

	Salt intake (g/24 h)	Iodine intake (mcg/24 h)	Iodine intake from food (mcg/24 h)	Iodine intake from salt (mcg/24 h)	Intake of iodized salt (g/24 h)	Intake of iodized salt as percent of total salt intake (%)
Children 3–9 years *n* = 614	7.0 ± 3.3	113.6 ± 78.2	83.0	30.6	1.46	21
Male adolescents (10–17 years) *n* = 349	9.5 ± 4.4	145.2 ± 89.4	94.5	50.7	2.41	25
Female adolescents (10–17 years) *n* = 307	8.8 ± 4.0	124.0 ± 89.9	88.6	35.4	1.69	19

The estimates suggest that the amount of iodine deriving from the use of iodized salt in all age and sex categories is lower than the amount provided by food, that this amount is higher for male teenagers because of their greater salt consumption and that the proportion of iodized salt intake compared with total salt intake is around 20%.

## Discussion

The data presented above indicate that:

1. Iodine intake increased gradually in our study population with increasing salt intake. This trend was independent of sex, age and body weight
2. The consumption of iodized salt was very low, corresponding to about one fifth of the total salt intake
3. A greater iodine intake and a lower probability of iodine inadequacy were achieved by a small proportion of the population examined, but this was associated with a very high salt intake
4. The majority of participants complying with the WHO recommendations of a total salt intake less than 5 g/day did not achieve an adequate iodine intake whatever the age group.

In addition, our data indicate that boys had a higher rate of iodine adequacy than girls, very likely as a consequence of their greater salt consumption. Also, salt consumption and iodine intake increased significantly with age (although iodine intake to a lower extent). Very likely, this increase was due to increased salty food consumption with age.

Few other studies focused on issues like those investigated in this report. A cross-sectional survey in Portuguese school children aimed to evaluate the iodine status and to monitor the use of iodized salt in school canteens and households [24]. The authors found a median urinary iodine concentration of 129 mcg/L in spot urine samples, but 32% of the examined children had low urinary iodine concentrations. The authors noted that no school canteen adhered to the “mandatory” use of iodized salt policy and only 2% of the households consumed iodized salt. They also found that lower consumption of milk, but not fish, was associated with a higher risk of iodine deficiency.

Charlton et al. [25] undertook a cross-sectional analysis of the WHO_SAGE Wave 2 Salt and Tobacco Study database to investigate to what extent the South Africa’s mandatory salt reduction policy affected its salt iodization program. In a random sample of adult population, they measured iodine and sodium excretion in 24 h as well as in spot urines. Similarly to our results, the authors found that iodine excretion increased with increasing salt consumption and that participants with salt consumption within the WHO limit of < 5 g/day did not meet the estimated average requirement (EAR) for iodine intake. The contribution of iodized salt to total salt and iodine intake was estimated by Esche and coworkers [26] in a large representative sample of German adult population (*Health-Interview and Examination-Survey for Adults, DEGS1, 2008–2011*) using spot urine collections. Results were compared with the published data from Switzerland. They estimated that only 42% of the total iodine intake was derived from salt, while in Switzerland it was 54%. The authors of both studies expressed concern that successful implementation of salt reduction policies will favor a decrease in iodine intake. He and coworkers carried out a randomized controlled trial of dietary salt reduction in a Chinese children population (the School-Edu Salt, School-based Education Programme to Reduce Salt) [27] to study the effect of salt reduction on iodine status, using 24 h urine collections. A 25% reduction in salt intake over three and half months was associated with a significant 19% reduction in iodine intake. Moreover, at the end of the trial there was a decrease in the proportion of children meeting the EAR for iodine: actually, only 5% of the sample had an iodine intake < EAR; however, it must be noted that universal salt iodization is mandatory in China and that iodized salt is regularly used by food manufacturers, thus as much as 80% of the total iodine intake is derived from salt. This value is much higher than that reported in Italy and in other European countries. In fact, the median baseline iodine intake of He’s study population was 165 mcg/day, i.e., over 50% larger than that of our Italian study population.

The Italian National Institute of Health (ISS) and the National Observatory for the Monitoring of Iodoprophylaxis (OSNAMI) in their 2014 Scientific Report focused on the role of salt iodization and the consumption of iodized salt to prevent iodine deficiency states (ISTISAN 2014) [10]. The authors measured the iodine content of several types of food representing major sources of dietary iodine and matched these results with the median values of consumption of the same foods in the pediatric and adolescent population based on the 2005-6 INRAN-SCAI survey [19]. They concluded that, given a median amount of approximately 70 mcg/day of iodine provided by food (mainly from dairy products and fish), an average intake of 5 g of salt per day (containing by law 30 mcg of iodine per g of salt) should be sufficient to meet the iodine adequacy level set at 150 mcg/day. The data of our report, obtained by the actual measurement of urinary iodine excretion in 24 h urines, indicate, however, that, in our study population, this was not the case. Indeed, a median estimated urinary iodine intake close to 150 mcg/day was achieved only in the subjects consuming more than10 g of salt a day. The discrepancy between expectations and actual data of iodine intake is explained by the finding that the proportion of iodized salt used in this nationwide sample of pediatric population was very low, i.e., around 20% of the total salt intake. The survey underwent in 2012 by Italian National Observatory for Monitoring Iodine Prophylaxis (OSNAMI) [10] showed that the percentage of iodized salt “sold” in Italy at that time was 50%: this amount of iodized salt obviously contributed to “discretionary” salt intake (i.e., salt added to the food while cooking or at table). According to Leclercq and Ferro-Luzzi [28], who carried a very careful study of the proportion of discretionary and non-discretionary salt intake by Italian households, discretionary salt represents between 35 and 45% of total salt intake in our country. If one half of this amount is iodized [10], the overall contribution of iodized salt to total salt intake becomes 17.5–22.5%. The remaining fraction of salt intake (“non-discretionary” intake) is given by salt that is naturally contained in food, which is not iodized (~ 10% of total salt) and salt added by the industry in the industrial food preparation (bread, pasta, pizza, sauces, etc. ~ 55–65% of total salt intake), which is by and large not iodized. In other words, when speaking of the percentage of iodized salt used by the population, one must refer to “total” salt intake not just to “discretionary” salt. If doing so, our estimates are fully compatible with the data from the OSNAMI survey which referred only to discretionary salt. This situation in Italy is the consequence of the fact that the Law n.55, 2005, is not compelling for the food industry with regard to the use of iodized salt but simply allows its use. We believe that this is a crucial weakness of the Italian legislation about iodoprophylaxis.

The relatively low use of iodized salt was not the only reason for low daily iodine intake. Indeed, a significant source of dietary iodine is represented by cow's milk and its derivatives, which have been demonstrated to be a major source of iodine in school age children [29]. It is thus possible that our study population had an even lower consumption of milk and dairies than predicted. Furthermore, since girls have been shown to consume less milk than boys [30] and given their lower consumption of total salt and, therefore, also iodized salt, this could help explain the significant difference in daily iodine intake between male and female participants.

We acknowledge as a limitation that our survey was carried out in 2012, while a more recent survey conducted in seven Italian regions on a sample of 11–13 y old school children has reported the use of iodized salt in 75% of the Italian school canteens [31]. On the other hand, no reliable evidence is available of a substantial increase in the use of iodized salt by households or of a higher proportion of iodized salt in processed foods. Indeed, in accordance with the current Italian legislation, all the salt retailers must ensure the availability of salt enriched with iodine but common edible salt is also available, including an increasing variety of fancy table salts (e.g., Kosher salt, Himalayan pink salt, Celtic sea salt, Fleur de sel), none of which is iodized. It is also prescribed that in the public catering sector iodized salt should be made available to consumers but not that it should be the “only” type of salt available. Finally, the use of salt fortified with iodine in food processing is permitted but not mandatory.

## Strengths and limitations of the study

Our work has several strengths as well as limitations. Major strengths are the use of 24-h urine samples to objectively measure salt and iodine intake, the relatively large size of our study population, and its widespread distribution across the Italian territory. Careful recommendations were given to ensure proper urine collection and the urines were checked for creatinine content in order to exclude from the analysis the individuals with creatinine excretion exceedingly low in relation to body weight. The major limitation is that, as acknowledged above, our study population was recruited 8 years ago so our results depict the situation at that time: nevertheless, we believe that these results are all the more important as no further survey has been carried out, since then the use of iodized salt by food manufacturers remains marginal and non-iodized salt is freely available in all food markets. A second limitation of our study is given by the fact that no dietary investigation was carried out to directly estimate the amount of iodine provided by food, so that we had to use estimates derived from other available national databases. A further limitation is that the household salt iodine content was not assessed. Finally, a limitation was the use of a single urine collection to estimate the participants’ habitual salt and iodine intake: it is known that repeated 24 h collections are necessary to estimate the “habitual” iodine intake of individuals with a high degree of accuracy [32]. For this reason, we only used the median iodine intakes of groups of participants with different salt consumption to depict the relationship between salt consumption and iodine intake. Median iodine intake for the participants in the age group 15–17 y with different levels of salt consumption (Table 2) could not be representative of because of the low number of participants in this age category.

## Conclusions

In conclusion, we observed that in our study population of Italian children and adolescents, adequate iodine intake was dependent on the consumption of large amounts of salt: this appears to be a consequence of (i) the limited consumption of iodine rich foods, as also suggested by the recent Health Behaviour in School-aged Children survey [33], (ii) the negligible amount of iodized salt provided by processed foods and (iii) the still insufficient use of iodized salt by households and by the catering system. It is particularly worrying that in our study population compliance with the WHO recommendations about salt intake reduction was associated with a very large percentage of iodine inadequacy. We believe that the results of our study are generalizable to the pediatric populations of other countries having legislations on iodoprophylaxis similar to the one currently in force in Italy. In keeping with the results of recent studies carried out in other countries [24–27], our data point to the need to continue to invest in vigorous policies encouraging the use of less salt but only iodized salt by citizens, by the catering system and by the food industry. In order for these policies to be successful, it is needed that the prescriptions contained in the current legislation (law 55/2005) are made compelling for all the involved stakeholders through specific enforcement measures that currently are lacking.

Additional strategies to prevent iodine deficiency could be an extended food and livestock fortification with iodine, the promotion of an increased consumption of iodine-rich foods (in particular milk, dairy products, and fish) and possibly an increase in the level of iodization of table salt, even if this third strategy could lead to exceed the tolerable upper intake of iodine. Finally, since the present results refer to a survey carried out several years ago, there is the need for a new national survey of the habitual sodium and iodine intake in the children and adolescent population in our country.

## Electronic supplementary material

Below is the link to the electronic supplementary material.

Supplementary file1 (DOCX 16 kb)

## Data Availability

All data generated or analysed during this study are included in this published article.

## Abbreviations

AI: Adequate intake
WHO: World Health Organization
EFSA: European Food Safety Authority
SIGENP: Italian Society for Pediatric Gastroenterology, Hepatology and Nutrition
UIC: Urine iodine concentration
BMI: Body mass index
IEO: European Institute of Oncology
INRAN: Istituto nazionale di ricerca per gli alimenti e la nutrizione
SCAI: Studi sui consumi alimentari in Italia
