# Supplementary material for: Relationship between salt consumption and iodine intake in a pediatric population
Source: Eur J Nutr. 2020 Oct 21;60(4):2193–202. doi: 10.1007/s00394-020-02407-w (PMC8137629; doi:10.1007/s00394-020-02407-w)
Supplement: Supplementary file 1 — Supplementary file1 (DOCX 16 kb) [file 394_2020_2407_MOESM1_ESM.docx]

**Supplementary Table**

Iodine content in food groups according to the WHO food composition tables [20], food mean consumption according to the INRAN-SCAI survey [19], and estimated iodine intake by food group (absolute and % of total). Note that the total iodine intake is reduced by 30% due to losses through cooking and inappropriate storage (see text).

a. Children (3-9 years old); b. Male adolescents (10-17 years old); c. Female adolescents (10-17 years old).

| **a.** | **Iodine content (mcg/100g)** | **Children mean consumption (g)** | **Iodine intake (mcg)** | **% contribution to iodine intake** |
| --- | --- | --- | --- | --- |
| Fish | 60 | 40.3 | 24.2 | 20.4 |
| Yogurt | 50 | 16.7 | 8.4 | 7.0 |
| Eggs | 50 | 20.0 | 10 | 8.4 |
| Cheese | 35 | 44.3 | 15.5 | 13.1 |
| Milk | 15 | 197.2 | 29.6 | 24.9 |
| Meat | 8 | 99.6 | 8.0 | 6.7 |
| Fruit | 5 | 136.0 | 6.8 | 5.7 |
| Cereals | 5 | 238.0 | 11.9 | 10.0 |
| Vegetables | 3 | 134.3 | 4.0 | 3.4 |
| Legumes | 3 | 10.1 | 0.3 | 0.3 |
| Total |  |  | 118.6 | 100 |
| **Actual iodine intake**  **(-30%)** |  |  | **83.0** |  |

| **b.** | **Iodine content (mcg/100g)** | **Male adolescents mean consumption (g)** | **Iodine intake (mcg)** | **% contribution to iodine intake** |
| --- | --- | --- | --- | --- |
| Fish | 60 | 48.4 | 29.0 | 21.5 |
| Yogurt | 50 | 13.5 | 6.8 | 5.0 |
| Eggs | 50 | 21.0 | 10.5 | 7.8 |
| Cheese | 35 | 63.8 | 22.3 | 16.5 |
| Milk | 15 | 168.3 | 25.2 | 18.7 |
| Meat | 8 | 145.1 | 11.6 | 8.6 |
| Fruit | 5 | 139.2 | 7.0 | 5.2 |
| Cereals | 5 | 331.3 | 16.6 | 12.3 |
| Vegetables | 3 | 186.3 | 5.6 | 4.1 |
| Legumes | 3 | 13.8 | 0.4 | 0.3 |
| Total |  |  | 135.0 | 100 |
| **Actual iodine intake**  **(-30%)** |  |  | **94.5** |  |

| **c.** | **Iodine content (mcg/100g)** | **Female adolescents mean consumption (g)** | **Iodine intake (mcg)** | **% contribution to iodine intake** |
| --- | --- | --- | --- | --- |
| Fish | 60 | 49.4 | 29.6 | 23.4 |
| Yogurt | 50 | 20.8 | 10.4 | 8.2 |
| Eggs | 50 | 20.8 | 10.4 | 8.2 |
| Cheese | 35 | 54.6 | 19.1 | 15.1 |
| Milk | 15 | 139.8 | 21.0 | 16.6 |
| Meat | 8 | 107.4 | 8.6 | 6.8 |
| Fruit | 5 | 178.5 | 8.9 | 7.1 |
| Cereals | 5 | 265.3 | 13.3 | 10.5 |
| Vegetables | 3 | 166.4 | 5.0 | 3.9 |
| Legumes | 3 | 9.5 | 0.3 | 0.2 |
| Total |  |  | 126.6 | 100 |
| **Actual iodine intake**  **(-30%)** |  |  | **88.6** |  |
